# MicroRNAs at the Crossroad of the Dichotomic Pathway Cell Death vs. Stemness in Neural Somatic and Cancer Stem Cells: Implications and Therapeutic Strategies

**DOI:** 10.3390/ijms21249630

**Published:** 2020-12-17

**Authors:** Andrea Diana, Giuseppe Gaido, Cristina Maxia, Daniela Murtas

**Affiliations:** 1 Department of Biomedical Sciences, University of Cagliari, 09042 Monserrato, Italy; 2 Cottolengo Mission Hospital Charia, Meru 60200, Kenya; fr.beppe@gmail.com

**Keywords:** miRNA, stemness, human neural stem cell, human neural/neural crest cancer stem cell, apoptosis, cell death, brain tumors, melanoma, neurodegeneration, therapeutic target

## Abstract

Stemness and apoptosis may highlight the dichotomy between regeneration and demise in the complex pathway proceeding from ontogenesis to the end of life. In the last few years, the concept has emerged that the same microRNAs (miRNAs) can be concurrently implicated in both apoptosis-related mechanisms and cell differentiation. Whether the differentiation process gives rise to the architecture of brain areas, any long-lasting perturbation of miRNA expression can be related to the occurrence of neurodevelopmental/neuropathological conditions. Moreover, as a consequence of neural stem cell (NSC) transformation to cancer stem cells (CSCs), the fine modulation of distinct miRNAs becomes necessary. This event implies controlling the expression of pro/anti-apoptotic target genes, which is crucial for the management of neural/neural crest-derived CSCs in brain tumors, neuroblastoma, and melanoma. From a translational point of view, the current progress on the emerging miRNA-based neuropathology therapeutic applications and antitumor strategies will be disclosed and their advantages and shortcomings discussed.

## 1. Introduction

From a semantic point of view, stemness and apoptosis may be conceived as two different sides of the same living pathway connecting ontogenesis to cell death, swinging from regeneration to demise. In this scenario, microRNAs (miRNAs) define a class of short length (up to 23 nucleotides) noncoding single-stranded RNA molecules acting as post-transcriptional regulators of gene expression, decisive for cell differentiation, survival, function, and dysfunction [1,2]. In a previous review, we highlighted the dramatic impact of the miRNA-target regulatory network both in human normal and tumoral neural stem cells (NSCs). In physiological conditions, not more than a dozen of miRNAs alternatively operate either for promotion or repression of neural induction. Similarly, in neural cancer stem cells (CSCs), miRNAs are recruited for a double goal: tumor suppressors or oncomiRNAs (oncomiRs) for the maintenance of CSCs and homeostasis of neuronal/glial tumors [3]. miRNAs modulate neural CSC apoptosis by inhibiting the expression of one or more target genes and by decreasing the level of the related translated proteins. If compared to non-tumor NSCs, neural CSCs show a significant downregulation of pro-apoptotic miRNAs, sometimes epigenetically inactivated by hypermethylation [4]. Upon restoration, these miRNAs can inhibit anti-apoptotic target genes, functioning as tumor-suppressive miRNAs. On the other hand, neural CSCs are characterized by the upregulation of anti-apoptotic miRNAs, which exert their control by inhibiting pro-apoptotic target pathways. These miRNAs are then considered as oncomiRs.

Moreover, the concept has emerged that the same miRNAs can be simultaneously involved in both apoptosis-related mechanisms and cell differentiation [5]. Whether the differentiation process gives rise to the architecture of brain areas, any long-lasting perturbation of miRNA expression could be related to neuropathological events. However, while the role of miRNAs in different forms of cell death has been exhaustively elucidated with regard to several organs and cancer progression [6,7], it still represents a critical task to investigate how miRNAs orchestrate cellular removal in human neurogenesis (i.e., corticogenesis) including those major niches of stemness of the adult brain, such as within the hippocampus and in the vicinity of lateral ventricles, providing that methodological tools are able to ascertain miRNA localization.

**Figure 1:**
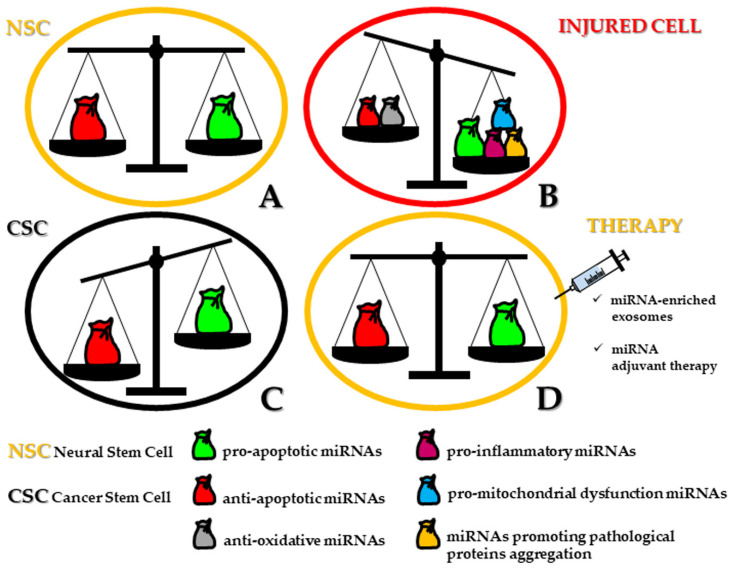
Contribution of different regulatory microRNAs (miRNAs) in the biology of neural stem cells (NSCs) (**A**), injured cells (**B**), cancer stem cells (CSCs) (**C**), and in therapy (**D**).

In this review, we aim to highlight recent key studies reporting the main specific miRNAs critically involved in the modulation of signaling pathways governing the balance among survival/self-renewal, differentiation, and cell death in human NSCs and neural/neural crest-derived CSCs (Figure 1). We will also discuss how these miRNAs may be promising as targets for therapeutic strategies in patients with neuropathologies or tumors of neural/neural crest origin.

## 2. miRNAs Bridging Stemness and Cell Death in Neurogenesis

Until now, embryonic and adult neurogenesis seem to share only miR-7 [8,9], -9 [10,11,12,13,14,15], -124 [16,17,18], and -137 [19,20] in the following functions: up or downregulation of proliferation, neuronal migration, axonal outgrowth/branching, neuron specification, and finally neuronal survival in the murine species (Figure 1A). Indeed, scant data are present in literature dealing with apoptosis-associated miRNAs for human neural differentiation and are obtained from human cell lines that show an association with modulated levels of miR-16, let-7a and miR-34a expression [21]. Concerning miR-34a, it has gained attention for the key regulatory role in neural stem/progenitor cell differentiation and various features of neurogenesis, but it is also implicated in a plethora of neurodegenerative events, such as Alzheimer’s (AD) and Parkinson’s (PD) disease, but also in epileptic seizures and ischemic stroke, suggesting its neuroprotective modulation especially when suppressed [22]. Interestingly, the inhibition of apoptosis in mature neurons, which is mediated by the induction of Bcl-2 homology 3 domain (BH3)-only proteins, is exclusively dictated by miR-29 upregulation [23]. Moreover, the survival of neural progenitor cells (NPCs) under hypoxia has been found to be determined by the upregulation of miR-210 by means of suppressed Bcl-2 interacting protein 3 (*BNIP3*), which is an activator of cell death [24].

On the subject of oxygen and glucose requirements, human NSCs have shown their vulnerability below a certain threshold of these nutrients, but the abundant cell death appearance was reduced by miR-21 overexpression through the inhibition of c-Jun N-terminal kinase (JNK) and p38 mitogen-activated protein kinase (p38MAPK) pathways.

However, when neuronal cell death takes place in an autophagic fashion, in hippocampal NSCs, miRNA expression profiling is complicated by the integrated functions of at least seven specific miRNAs (miR-34b-5p, -138-5p, -351-5p, -503-5p, -542-5p) that are actively involved under insulin-deprived conditions. Therefore, during senescence, because of the limited replacement of dysfunctional neurons, the necessary goal of the neural network within the whole brain is attained by reversing or circumventing the apoptotic pathway, ultimately responsible for the symptomatic manifestation of various age-related diseases. On this matter, dysregulated miRNAs have been described in recent years as possible additional inducers of cellular stress and related vulnerability for neurons and, therefore, implicated in several neurodegenerative disorders [25] (Figure 1B).

It exceeds the purpose of this paper to describe the functions and potential applications of the numerous miRNAs in terms of diagnostic biomarkers since comprehensive reviews have been already written for AD [26,27,28] and PD [29,30,31], amyotrophic lateral sclerosis (ALS) [29,30,31,32], multiple sclerosis (MS) [33,34], traumatic brain injury (TBI) [35,36], or development-related syndromes, such as autism spectrum disorder (ASD) [37,38]. Instead, both in the central nervous system (CNS) and in the cerebral spinal fluid (CSF), miRNAs recruitment has boosted a novel impulse for the prognosis but, more strikingly, for neural regenerative medicine, shedding light on feasible translational brain applications that fill the present gap of clinical interventions represented by NSC transplantation.

### miRNA-Loaded Exosomes for Therapeutic Interventions in Neuropathologies

Now, it is well established that during the formation of the neural network in the embryonic period and when neural regeneration occurs, neural cells release extracellular vesicles, also named exosomes, as an alternative intercellular communication beyond the classical synaptic neurotransmission. The decisive discovery has come from the observation that such exosomes are secreted by cortical neurons [39] and represent an efficient delivery method for integrating different miRNAs into the target neural cells [40,41,42,43]. Therefore, miRNAs have gained further attention because of their property to be inserted into mesenchymal stem cells (MSCs) or human induced pluripotent stem cells (hiPSCs) and to be released with a paracrine function or transported in order to follow an endocytic pathway [44].

MiRNA-associated exosomes display some advantages when isolated from the original cells, which can be enunciated as follows: first of all, the ability to cross the blood–brain barrier, inducing a minimal tumorigenic and immunogenic response but with a half-life longer in transplanted patients, and finally being insensitive with regard to proliferation and microvascular embolism. Collectively, exosomes, miRNAs and stem cells seem to depict the tiles of a puzzle defining the Holy Grail for therapeutic intervention.

As a matter of fact, the above properties have proposed that trio as the most promising candidate for the treatment of neurological diseases. Starting from the most challenging AD, the efficacy of treating neural degeneration has been evaluated in vivo in an amyloid β-peptide (Aβ)-treated rat model that was transplanted with packaged miR-29-engineered exosomes using transfected MSCs. The final result was the prevention of spatial and learning deficits typical of AD [45]. For in vitro experiments [46], the extracellular vesicles isolated from hiPSCs and enriched with miR-133, -155, -221 and -34a conferred neuroprotection to Aβ42 oligomer-treated cortical spheroids [47,48]. With regard to remyelination strategy, which could positively impact MS, rat environmental enriched exosomes have been found to be upregulated in miR-219 content, fostering the increase in myelin but reducing the observed oxidative stress because of the dramatic consequences on the number of oligodendrocyte precursors and stem cells [49].

The therapeutic efficacy and safety of exosomes and inserted miRNAs have also been evaluated for stroke, although only a few clinical trials have been conducted in stroke patients. So far, rat stroke models and NSCs derived from different tissues have been used. Patients’ plasma has been exploited for demonstrating that miR-126 is downregulated when acute ischemic stroke (AIS) occurs as well as in rat plasma and brain tissue after ischemia. Following miR-126 enrichment of adipose-derived stem cells (ADSCs)-purified exosomes, a significant reduction in neuronal cell death accompanied by a higher proliferation rate was observed. In addition, the miR-126-modified exosomes induced, in the post-stroke period, a functional recovery accompanied by a clear suppression of the inflammatory cascade [50].

Some more studies have identified in the multipotent MSCs the favorite tool for the production of miRNA-133b-bearing exosomes in rats subjected to middle cerebral artery occlusion (MCAO). In this context, the relevant contribution of astrocytes by the secretion of exosomes containing miR-133b in trophic activity for the neuronal plasticity in terms of neurite branching and elongation has been elucidated [51].

The same authors have further shown that miR-17-92 cluster-enriched exosome treatment produces a boosting effect on neurological functions by recruiting new neurons and oligodendrocytes, meanwhile determining an overall remodeling of neuronal fibers [52].

In a different experimental setting, performing administration of MSC-miR-146a-5p-exosomes, there was a confirmation of restored neurological functions, and reduced neuronal demise was associated with the inhibition of microglia in terms of M1 polarization, which accounted for the decreased expression of some pro-inflammatory mediators [53].

When NSCs were subjected to oxygen–glucose deprivation/reoxygenation for mimicking the ischemic stroke manifestation, the miR-26a exosomes from human urine-derived stem cells were able to promote both proliferation and neuronal differentiation, revealing an alternative strategy to hinder brain ischemia [54].

However, most of the available data in literature dealing with exosomes and miRNAs refer to traumatic brain and spinal cord injury (SCI). Recently, some scientists have focused on the involvement of microglial exosomes carrying miR-124-3p as cargo, whose beneficial effects were described as neuronal survival by switching M2 polarization in microglia and therefore inhibiting neuroinflammation [55]. Further evidence has been provided by showing that exosome-associated miR-124 administration increased hippocampal neurogenesis and functional recovery in the same way by silencing the Toll-like receptor 4 (TLR4) pathway [56]. In addition, the neurite outgrowth could be ascribed to microglial-derived exosomes enriched in miR-124-3p and entailing neuronal autophagy [57]. Further, repetitive mild traumatic brain injury (rmTBI) has been thought as an important risk factor for long-term neurodegenerative disorders such as AD, where microglial exosomes have exhibited a cardinal role in the transportation, distribution, and clearance of Aβ. In particular, the miR-124-3p level in microglial exosomes from an injured brain was significantly changed in the acute, sub-acute, and chronic phases after rmTBI. The effects were accomplished by miR-124-3p targeting RELA proto-oncogene, NF-KB p65 subunit (*RELA*), an inhibitory transcription factor of apolipoprotein E (ApoE) that endorses β-amyloid proteolytic breakdown. In mice with rmTBI, neurons of injured brains could internalize the intravenously injected microglial exosomes and ultimately gave a robust contribution to prevent neuronal death [58].

In patients with SCI, relevant evidence for the recovery of neurological functions has been reported with regard to the positive modulation of miR-21 derived from the exosomes of MSCs [59]. This last finding has been also confirmed by a paper in which not only miR-21 but also miR-19b was found to be increased in extracellular vesicles from isolated MSCs and consequently were effective in apoptosis inhibition by means of downregulation of phosphatase and tensin homolog (*PTEN*) messenger RNA (mRNA) [60]. The mechanism by which MSC-derived exosomes carry out their neuroprotective effects on motor function and apoptosis has been found following the modulation of Fas cell surface death receptor ligand (*FASL*) gene axis [61].

miR-133b has been recognized as an important actor for the differentiation of neurons and the outgrowth of neurites. In this perspective, exosomes derived from miR-133b-modified MSCs have been found to induce recovery of hindlimb function after injection in rats with SCI, by activation of the extracellular regulated kinase 1/2 (ERK1/2), signal transducer and activator of transcription 3 (STAT3), and cAMP response element-binding protein (CREB) pathways [62]. These results were replicated by a study where, in addition to the significative increase in those genes, neurofilament (*NF*), growth associated protein 43 (*GAP43*), glial fibrillary acidic protein (*GFAP*), and myelin basic protein (*MBP*) were also upregulated in SCI rats [63].

Exosomes from miR-29b-modified MSCs were also able to repair SCI when injected in rats with an increased score for hindlimb motor function [64].

Finally, exosomal miR-544 derived from bone marrow mesenchymal stem cells (BMSCs) mitigated neuronal functional recovery after SCI. Moreover, overexpression of miR-544 in BMSC exosomes attenuated histologic deficits and neuronal cell death induced by SCI. Remarkably, this therapeutic intervention also abated inflammatory events following SCI. In conclusion, exosomes derived from miR-544-overexpressing BMSCs improved functional recovery and promoted neuronal survival by attenuating inflammation after SCI [65].

## 3. miRNAs Involved in the Regulation of Apoptosis in Human Neural/Neural Crest-Derived CSCs

Along with regulating several biophysiological processes within NSCs, miRNAs are critically responsible for the control of cell fate in neural CSCs harbored in tumors of both neural and neural crest origin. These CSCs, considering the core cell subset of these tumors, express the NSC lineage markers nestin, prominin-1 (CD133), nanog homeobox (NANOG), sex determining region Y-box 2 (SOX2), hematopoietic cell E/L-selectin ligand (HCELL/CD44), octamer-binding transcription factor 4 (OCT4), bear a marked ability to form neurospheres in particular conditions [47,48], and are characterized by a tumor-initiating and self-renewal capacity through asymmetric cell division [3,66,67,68]. Moreover, the hallmarks of CSCs include high proliferation rate, multi-lineage differentiation potential, as well as great tendency for cell migration, invasiveness, and metastasis [69,70,71]. The CSC phenotype is also characterized by an enhanced resistance to apoptosis, a mechanism exploited to eliminate dysfunctional or genotoxic stress-exposed cells [4] (Figure 1C).

The tumor protein p53 (*TP53*) gene encodes for the key transcription factor, which coordinates cell responses to cancer-initiating insults, such as DNA damage and oncogene activation. When activated, p53 regulates the expression of a wide set of target genes promoting apoptosis, senescence, cell cycle arrest, and DNA repair [72,73,74,75]. Neural CSCs can escape apoptosis either by the downregulation of death receptors, such as death receptor 5 (*DR5*) and its tumor necrosis factor apoptosis inducing ligand (*TRAIL*), or by the overexpression of anti-apoptotic factors, such as cellular FLICE-like inhibitory protein (*c-FLIP*), Bcl-2 apoptosis regulator (*Bcl-2*) family members, BMI1 proto-oncogene, polycomb ring finger *(BMI-1*), nuclear factor kappa-light-chain-enhancer of activated B cells (*NF-κB*), inhibitors of apoptosis proteins (IAPs), as well as by the aberrant upregulation of phosphoinositide-3-kinase/serine-threonine protein kinase/mammalian target of rapamycin (PI3K/AKT/mTOR) signaling. The ratio between pro- and anti-apoptotic contents determines the balance between cell survival or cell death and modulates the susceptibility of CSCs to undergo apoptosis [76,77].

Recent in vitro and in vivo studies have shown that most neural/neural crest-derived CSCs, including brain tumor stem cells (BTSCs), glioma stem cells (GSCs), medulloblastoma (MB) as well as neuroblastoma (NB) and melanoma stem cells, are resistant to standard antitumor treatments, i.e., chemotherapy and radiotherapy, mainly due to high expression of anti-apoptotic genes, ATP binding cassette transporter (ABC) family multidrug resistance transporters, and by rapid activation of DNA damage response pathways. Additionally, neural CSCs survival despite therapy and their following rapid self-renewal may result in the recurrence of the above-mentioned cancers [70,76,78,79,80,81].

Herein, we will report the miRNAs, described so far, which bear the potential to fine-tune the expression of genes intercalated in signaling networks regulating human neural/neural crest-derived CSC apoptosis, guiding the outcome of the whole tumor (Table 1). If a neural CSC is addressed to self-renewal—thus sustaining the tumor—or not critically depends on its miRNA expression profile and, ultimately, on the miRNA-targeted genes [82].

Distinct miRNAs play a key role in interfering with the apoptosis machinery by targeting p53-related genes. On the other hand, p53 itself regulates CSC properties through the modulation of miRNA expression. A cohort of miRNAs exhibits p53-dependent regulation following DNA damage. As an example, miR-34a is a component of the p53 tumor suppressor network, where miR-34a and p53 cooperate to orchestrate cell cycle and apoptosis signaling. miR-34 transactivation by p53 results in a dramatic reprogramming of gene expression, which ultimately promotes apoptosis [89,115,216,217,218]. Furthermore, miRNAs are able to induce drug resistance by targeting key cell cycle regulatory genes, such as cyclin-dependent kinase 6 (*CDK6*), retinoblastoma protein (*RB*), E2 factor family of DNA-binding transcription factors (*E2F*), and cyclins that initiate S-phase [93].

Since miRNAs can target multiple genes and their expression pattern and function are influenced by cell- and tumor-specific environmental factors, specific miRNAs can exert opposite activities in different cancers. For this reason, the identification of differentially expressed miRNAs and their context-dependent target genes seems to be of great help in discovering the network of signaling pathways boosting specific tumors [219].

As reported in our recent review, miRNAs can act as either onco-suppressive or oncomiRs by directly regulating the commitment to apoptosis and the cell cycle progression of neural CSCs housed in brain tumors, such as glioblastoma multiforme (GBM) and MB [3].

Glioblastoma multiforme, the maximal progression stage of glioma, is the most frequent and deadliest adult CNS tumor, arising from diverse cell lineages in the adult brain, comprising NSCs, NSC-derived astrocytes, and oligodendrocyte precursor cells (OPCs), due to the dysregulation of transcriptional and epigenetic networks governing self-renewal and differentiation of these cells [136,220,221,222].

Medulloblastoma is the most common childhood brain tumor, originating from the aberrant proliferation of granule-cell progenitors and NSCs during development of the cerebellum [90,223].

Both of these cancers are characterized by a high cellular heterogeneity, with a constitutive CD133+/CD15+ (stage specific embryonic antigen 1, SSEA-1) neural CSC subpopulation feeding the bulk tumor [71,223,224,225]. GSCs are the most studied neural CSCs. They are considered glioma-initiating cells and are crucial for the malignancy and recurrence of glioma. Due to their chemo- and radioresistant behavior, there is a pressing need to better understand the biology of these CSCs, so that therapy could benefit from their targeting and destruction. In the last few decades, studies investigating the dysregulated expression of miRNAs in these CSCs have shed considerable light onto the critical processes supporting the pathogenesis and progression of such malignancies [136,226,227,228,229,230,231].

Neuroblastoma is an extracranial pediatric neuroendocrine tumor, originating from embryonal or fetal neural crest stem cells, destined to become the sympathetic ganglia of the autonomic nervous system or the catecholamine-secreting cells of the adrenal glands. It is well established that epigenetic modulation, associated with developmental reprogramming errors, may drive the genesis of pediatric cancers, such as NB [232]. Its origin from pluripotent progenitors confers to NB a high level of heterogeneity and a hindrance to differentiate, which often impedes the success of antitumor therapy [233,234]. As other tumors, NB houses a CSC population, responsible for its aggressive nature [163]. Some miRNAs have been demonstrated to be differentially expressed in NB CSCs, with miRNA profiles being strongly associated with apoptosis management [112,212,234].

Melanoma is a highly aggressive and deadly neuroectodermal tumor developing by the malignant transformation of neural crest-derived melanocytes. Due to its frequency and aggressiveness, it represents the most thoroughly studied neural crest-derived cancer [235]. In the bulge area of dermal hair follicles, a source of melanocyte stem cells (MCSCs) has been localized, which, if genetically or epigenetically altered, can proliferate and migrate abnormally, acting as melanoma cells of origin in the skin and resulting in melanomagenesis [236,237]. Melanoma harbors high cell heterogeneity including multi-subpopulations of cancer cells, some of which, due to their stemness features, are identified as melanoma stem cells and can be detected by the neural cell surface marker CD133, as well as by the neuroectodermal internal marker nestin [71,79,238,239,240,241].

### 3.1. Pro-Apoptotic miRNAs

Diverse miRNAs with pro-apoptotic function have been identified so far and their downregulation in neural/neural crest CSCs has been described as a factor promoting tumorigenesis.

miR-7 expression is significantly diminished in GSCs, which grants such cells strong resistance to apoptosis. However, by engineering a panel of GSCs to express miR-7, the role of this miRNA in targeting the apoptotic pathways in GSCs has been revealed, by means of downregulated expression of epidermal growth factor receptor (*EGFR*), suppressed AKT signaling activation, and upregulated *DR5*; thus, sensitizing GSCs to TRAIL-mediated apoptosis [83].

miR-23b is epigenetically silenced by hypermethylation in U87 GSCs, where the apoptotic machinery results in being suppressed. When this cell subpopulation is transfected with miR-23b mimics, it downregulates its target gene high mobility group AT-hook 2 (*HMGA2*) and, meanwhile, sensitizes U87 GSCs to temozolomide (TMZ)-induced apoptosis [84].

miR-26a is significantly downregulated in A375 melanoma stem-like cells, which endow a subpopulation with CSC-like traits, characterized by the ability to form melanospheres [203,242]. This miRNA has been shown to induce apoptosis in A375 cells by directly targeting the silencer of death domains (*SODDs*) gene, which has a pivotal role in halting apoptosis in sensitive melanoma cell lines [85,86].

miR-29a is also reduced in CD133+ GSCs, allowing GSCs to have continuous activation of survival-promoting pathways. When ectopically expressed, miR29a targets Quaking gene isoform 6 (*QKI-6*), which leads to the Wilms tumor protein 1 (WT1)-mediated repression of key pro-survival target genes, such as *EGFR* [87]. Moreover, miR-29a overexpression sensitizes CD133+ GSCs to cisplatin-induced apoptosis [88].

miR-34a is downregulated in GSCs and MB CSCs, resulting in increased cell survival and reduced CSC differentiation [91]. Conversely, miR-34a experimental induction in U87 GSCs inhibits the expression of the *Bcl-2* as well as the Notch receptor 1/2 (*NOTCH1/NOTCH2*) and Notch ligand Delta-like 1 (*Dll1*) genes, boosting apoptosis and cell differentiation. According to cell cycle analysis, ectopic miR-34a expression induces cell cycle arrest in G1 phase [3,90,92,105]. In HNGC-2 and NSG-K16 GSCs, restored miR-34a functionally targets the rapamycin-insensitive companion of mTOR (*RICTOR*) gene, a defining component of the mammalian target of rapamycin complex 2 (mTORC2); thus, suppressing the AKT and wingless-related integration site (Wnt)/β-catenin signaling pathways and reducing the levels of the Wnt pathway-target proteins cyclin D1 (CCND1) and myc proto-oncogene (c-MYC) [219].

In A375 melanoma stem-like cells, miR-34a expression is silenced by aberrant methylation of its promoter, which can provide a selective benefit for tumor cells by suppressing the p53 machinery and activating the Notch signaling. However, miR-34 expression is restored after etoposide treatment, which results in increased apoptosis and cell cycle arrest, thereby recovering the effect of p53. Thus, these findings may explain, at least in part, the anti-proliferative effect of miR-34a in melanoma stem-like cells [79,93,94,95].

miR-107 expression is diminished in GSCs. When re-expressed, this miRNA functions as a tumor suppressor gene and inhibits CD133+ GSCs proliferation by targeting the *NOTCH2* and matrix metalloproteinase-12 (*MMP-12*) mRNA expression [96].

miR-124 and miR-137, simultaneously lost along gliomagenesis, when restored, induce G0/G1 cell cycle arrest of GSCs, in association with decreased expression of CDK6 and phosphorylated RB (pSer 807/811) proteins [4,97,98,100,105]. miR-137 also downregulates the mRNA and protein level of glioma pathogenesis-related protein (*GLIPR*), also known as related to testis-specific, vespid, and pathogenesis proteins 1 (*RTVP-1*), in GSCs; thus, rescuing cell responsiveness to apoptotic effects [99].

miR-137, located at a melanoma hotspot in the human genome, is significantly decreased in A375 melanoma stem-like cells. It can act as a tumor suppressor by directly targeting C-terminal binding protein 1 (*CTBP1*), inhibiting epithelial-to-mesenchymal transition (EMT) and inducing apoptosis [100]. In these cells, miR-137 has also been shown to be a negative regulator of phosphatidylinositol-3-kinase regulatory subunit 3 (*PIK3R3*), a transducer of intracellular lipid substrates that is involved in the regulation of apoptosis [101,102].

miR-125a-5p is downregulated in A375 melanoma stem-like cells. Although this condition accelerates cell growth in vitro, when re-expressed, miR-125a-5p functions as a tumor suppressor by directly targeting lin-28 homolog B (*LIN28B*), which serves as its functional effector. Furthermore, the involvement of a let-7-dependent mechanism downstream of LIN28B has been demonstrated, which results in the activation of the transforming growth factor-β (TGF-β) signaling cascade [103,104,140].

miR-125b is amongst the first miRNAs described as downregulated in human U251 GSCs, with a pivotal role in GSC maintenance. In CD133+ GSCs, the downregulation of miR-125b leads to E2F transcription factor 2 (*E2F2*) expression and cell cycle progression. When its functionality is restored, miR-125b suppresses the expression of cell cycle regulatory proteins CDK6, a G1/S cell cycle regulator, and cell division cycle 25A (CDC25A), thereby inducing cell cycle arrest at the G1/S checkpoint [105,106,107,108]. Nonetheless, emerging evidence has further demonstrated that miR-125b can be overexpressed in GSCs, where it targets tumor necrosis factor alpha-induced protein 3 (*TNFAIP3*) and NF-κB inhibitor interacting Ras-like 2 (*NKIRAS2*), *TP53*, *p38MAPK*, resulting in increased NF-κB expression, anti-apoptotic activity, and upregulation of cell cycle genes [109]. This relates to the resistance of GSCs to tumor necrosis factor alpha (TNFα)- and TMZ-induced apoptosis; thus, classifying miR-125b as a predictor of TMZ response in GBM patients. Similarly, miR-125b enhances TMZ resistance by targeting Bcl-2 antagonist/killer 1 (*BAK1*) [110]. The repression of miR125b before TMZ treatment improves the chemosensitivity of GSCs to TMZ and thus results in inhibition of cell proliferation and increased apoptosis [111]. The double function of miR-125b in tumor regulation indicates that it may act as a potent tumor promoter or inhibitor depending on the different molecular context. Considering that a miRNA can target more than one gene, it would be deduced that the panel of genes that predominantly contribute to the phenotypes prompted by the miRNA may depend on the cell microenvironment. In this context, further studies will hopefully elucidate the dual role of miR-125b, supporting its value as therapeutic target.

In NB, the knowledge regarding the expression of tumor suppressive miRNAs or oncomiRs in CSCs is still limited. One of the few reported studies has shown that miR-125b may act as an oncogene in N-type SH-SY5Y human NB cells, which have many similarities to CSCs, such as the expression of the neuroectodermal stem cell intermediate filament nestin and the capacity to form colonies in soft agar [243,244,245]. Overexpression of miR-125b is a negative regulator of *TP53* and p53-induced apoptosis in NB cells. Conversely, knockdown of miR-125b has been demonstrated to retrieve the level of p53 protein and induce apoptosis [112,113].

In A375 melanoma stem-like cells, miRNA expression profiling has shown an overexpression of miR-125b, -100, and -199-5p, while miR-513a-5p and -185 are underexpressed, with miR-125b being considered as the determinant candidate of melanoma progression, through its ability to target the neural precursor cell expressed, developmentally down-regulated 9 (*NEDD9*) gene [114].

miR-128 is remarkably reduced in CD133+ GSCs, favoring GSC maintenance and tumor growth. However, upon re-expression, it represses GSCs’ growth, mediates their differentiation, and exerts a pro-apoptotic role by downregulating the oncogenic EGFR/platelet-derived growth factor receptor/AKT (EGFR/PDGFR/AKT) pathway and *BMI-1*, a factor critical for an efficient self-renewing division of GSCs [105,115,116,117].

*BMI-1* is also targeted by the onco-suppressor miR-218, commonly downregulated in GSCs [3,105,108,118]. The mechanisms by which exogenous administration of miR-218 rescues apoptosis in U87 GSCs also includes the functional targeting of *CDK6*, marker of proliferation Ki-67 (*MKI67*), and epidermal growth factor receptor-coamplified and overexpressed protein (*ECOP*), which affects NF-κB activity and the associated apoptotic response [119,120].

miR-129-5p expression is downregulated in U87 and U251 GSCs, with fibronectin type III domain containing 3B (*FNDC3B*) as a target gene. U87 cells transfected with miR-129-5p mimics and FNDC3B short hairpin RNAs (shRNAs) exhibit restored cell apoptosis, corroborating the tumor-suppressing role of miR-129-5p [121].

miR-134 expression is lost in BTSCs and in sphere-forming U87 GSCs, causing inhibition of apoptosis and uncontrolled cell proliferation. Conversely, its ectopic expression promotes apoptosis and cell differentiation by regulating *NANOG* as the main target, at the mRNA and protein level [122].

miR-138 expression level is downregulated in A375 melanoma cells. When ectopically overexpressed, it increases apoptosis and inhibits cell proliferation and metastasis by directly targeting the degradation of hypoxia-inducible factor-1 alpha (*HIF1α*). Therefore, it functions as a suppressor of melanoma occurrence and development [123].

miR-141 expression levels are greatly reduced into the CD133+ GSC subpopulation, conferring resistance to apoptosis. However, following overexpression in GSCs, miR-141 can inhibit the jagged canonical Notch ligand 1 (*JAG1*) and thus restoring the expression of apoptosis-related genes [124].

miR-145 is expressed at very low levels in GSCs, which is associated with increased tumorigenesis. Though, when experimentally induced, it increases U87 GSC apoptosis by inhibiting BNIP3 and Notch signaling [125,126]. Moreover, targeting of SRY-box transcription factor 9 (*SOX9*) and adducin 3 (*ADD3*) by miR-145 has been reported, consistent with increased apoptosis [127].

In melanoma cell lines, miR-145 has been proposed as a miRNA whose reduced expression implies the acquisition of a stem-like phenotype, defined by the ability to grow non-adherently as melanospheres in vitro [115,246].

miR-146b-5p expression is lowered in gliomagenesis. However, miR-146b-5p overexpression, by suppressing Hu antigen R (*HuR*) expression, increases apoptosis and radiosensitivity, and induces differentiation in GSCs [128].

miR-149 underexpression has been demonstrated in SK-N-BE(2)-C and SK-N-SH NB stem-like cells and in NB tissues, and is associated with poor survival rates. However, transfection of these cells with miR-149 re-establishes its pro-apoptotic and anti-proliferative function by targeting *Bcl-2* and cell division cycle 42 (*CDC42*). Furthermore, introduction of miR-149 increases chemosensitivity to doxorubicin (DOX) in NB cells [129].

miR-152 and -153 expression levels are reduced in cultured CD133+ GSCs and are critically involved in glioma tumor growth. Re-expression of these two miRNAs reduces cell proliferation and induces differentiation and apoptosis by downregulating the transcription factor Krüppel-like factor 4 (*KLF4*), inhibiting the expression of galectin 3 (*LGALS3*), and indirectly attenuating the activation of mitogen-activated protein kinase kinase 1/2 (MEK1/2) and PI3K signaling pathways [3,105,130,131,132].

Reduced miR-181 expression in GSCs has been demonstrated to upregulate *CD133* expression and the downstream signaling pathways. Instead, restoration of miR-181, by targeting the *NOTCH2* receptor gene, downregulates CD133 and retrieves cell apoptosis [133]. Moreover, transient overexpression of miR-181a in U87 GSCs significantly sensitizes these cells to radiation treatment, concurrently with downregulation of the Bcl-2 protein [134].

Endogenous miR-182 levels are decreased in U87 GSCs, leading to GBM growth, hypoxia-induced dedifferentiation, and tumor progression. However, ectopic expression of miR-182 negatively regulates the oncogenic signature, including Bcl-2 family protein Bcl-2-like protein 12 (*BCL2L12*), MET proto-oncogene, receptor tyrosine kinase (*c-MET*), and hypoxia-inducible factor-2 alpha (*HIF2α*). Conversely, miR-96 and miR-183, also belonging to the 183-96-182 miR cluster, fail to crack down on the oncogene *BCL2L12* expression, which is able to neutralize effector caspases and p53 activation. Moreover, miR-182 sensitizes glioma cells to therapy-induced apoptosis [135].

In CD15+/CD133+ MB cells, expression of miR-199b-5p is downregulated by class B basic helix-loop-helix protein 39 (*HES1*) overexpression, with a negative feedback regulation, and by methylation of the cytosine-phosphate-guanine (CpG) island upstream of its promoter region. On the other hand, when overexpressed, miR-199b-5p can reduce the subpopulation of MB CSCs and induce cell differentiation, with *HES1* as a target gene involved in both the canonical Notch and noncanonical sonic hedgehog (SHH) pathways [117,224].

miR-200b, a member of the miR-200 family, is significantly decreased in glioma U251 cells. Cell transfection with miR-200b mimics, on the contrary, results in decreased expression of *CD133* mRNA and restored cell apoptosis [137].

Downregulation of miR-203, as a tumor suppressor, is responsible for the maintenance of stem properties of CD133+ GSCs. When re-expressed, miR-203 can retrieve apoptosis by directly repressing its target phospholipase D2 (*PLD2*) [138].

On the contrary, miR-203 has been shown to be overexpressed in melanospheres derived from D10 and A375 melanoma stem-like cells, displaying its dual effect observed in several cancers. In these cells, miR-203 enhances the number and size of colonies and leads to the upregulation of *SOX2*, *KLF4*, and *OCT4*, as main transcription factors in the management of pluripotency [247].

miR-211 is greatly decreased in GSCs by aberrant hypermethylation of its promoter region. After rescuing its expression, miR-211 downregulates the expression of matrix metalloproteinase-9 (MMP-9) and anti-apoptotic proteins, such as Bcl-2. At the same time, the levels of cell cycle inhibitors, such as p53, and the proteolytic activity of caspase-9 (CASP9), increase; thus, triggering the mitochondrial/caspase-3 (CASP3)/CASP9-mediated apoptotic pathway [139].

Another target of miR-211 is potassium calcium-activated channel subfamily M alpha 1 (*KCNMA1*), overexpressed in A375 melanoma stem-like cells due to the endogenous low levels of miR-211. When this miRNA is ectopically expressed, these cells face significant growth inhibition, compared with the respective parental melanoma cell lines [140,141,142,143].

miR-218 is a tumor suppressive miRNA decreased in A375 melanoma stem-like cells, while the transfection of these cells with this miRNA mimic has been shown to cause cell cycle arrest in the G0/G1 phase, by targeting cellular inhibitor of PP2A (*CIP2A*) and *BMI-1*, supporting the hypothesis of the pivotal role of miR-218 in melanoma development [102,144].

miR-219-5p is downregulated in A375 melanoma stem-like cells, negatively correlating with Bcl-2 protein levels. In these cells, the transfection of miR-219-5p restores intrinsic apoptosis by suppressing the antiapoptotic gene *Bcl-2* and increasing cleaved CASP3 and CASP9 levels. Thus, the upregulation of miR-219-5p inhibits melanoma growth and metastasis and strengthens melanoma cells’ chemosensitivity [145].

The miR 302-367 cluster is undetectable in glioma-initiating cell lines. However, during serum-mediated stemness suppression, its induced expression leads to a drastic downregulation of C-X-C motif chemokine receptor 4 (*CXCR4*) and its ligand C-X-C motif chemokine ligand 12 (*CXCL12*), which impairs the stemness characteristics and induces differentiation of GSCs [146].

miR-326 is downregulated, via decreased expression of its host gene, in GSCs and in D283 Med CSCs. Transfection of miR-326 into GSCs can rescue its functionality, inhibiting the Notch signaling pathway and impairing cell proliferation rate. NOTCH, in turn, is able to suppress miR-326, establishing a regulatory feedback loop. The NOTCH/miR-326 axis in GSCs is shifted towards high NOTCH and low miR-326 activity, but it can be reversed by miR-326 transfection [108,117,147]. Moreover, miR-326 ectopic expression represses smoothened, frizzled class receptor (*SMO*) and its downstream gene glioma-associated oncogene homolog 1 (zinc finger protein) (*GLI1*), which is no longer able to translocate into the nucleus and to regulate its target genes’ transcription [148,149,150].

miR-340 is decreased in U87 and U251 GSCs, triggering GBM development. Conversely, reintroduction of miR-340 leads to inhibition of cell proliferation and induction of apoptosis by directly targeting Rho associated coiled-coil containing protein kinase 1 (*ROCK1*) [151].

Expression of miR-451 has been found to be significantly reduced in U251 GSCs, impacting their survival and proliferation rate. On the contrary, administration of miR-451 mimic oligonucleotides results in G0/G1 phase cell cycle arrest and increased apoptosis, through *AKT1*, *CCND1*, matrix metalloproteinase-2 (*MMP-2*), *MMP-9*, and *Bcl-2* reduction, and increase in cyclin-dependent kinase inhibitor 1B (*CDKN1B/P27^KIP1^*) expression. Further evidence has suggested that the regulation of miR-451 by the mothers against decapentaplegic homolog (SMAD) protein, when transfected in GSCs, inhibits cell growth, likely by the upregulation of miR-451 [152,153]. Moreover, miR-451 represses U251 GSC proliferation and induces apoptosis, by regulating calcium binding protein 39 (*CAB39*) as a direct target and indirectly inhibiting the PI3K/AKT pathway [154].

miR-503 is also subject to downregulation in U87 and U251 GSCs, a condition that prompts cell proliferation. When ectopically induced, miR-503 triggers apoptosis and leads to G0/G1 cell cycle arrest, by negatively regulating insulin-like growth factor-1 receptor (IGF-1R) and consequently weakening the functionality of the IGF-1R/PI3K/AKT signaling pathway, a core pathway in GBM oncogenesis [155].

Downregulation of miR-608 has been recently linked to an increase in the macrophage migration inhibitory factor (*MIF*) gene and protein in GSCs. On the contrary, restored miR-608 expression negatively regulates the expression of *MIF* and induces apoptosis in GSCs [3,156].

Expression level of miR-625 is significantly low in A375 melanoma cells, although it can be recovered upon cell transfection with miR-625 mimics, which results in enhanced apoptosis and inhibited cell growth, by the silencing of the transcription factor *SOX2* [104,157].

miR-873 is expressed at low levels in U87 GSCs, playing a pivotal role in GBM development. Restoration of miR-873 inhibits cell proliferation and induces apoptosis, by directly knocking down insulin-like growth factor 2 MRNA binding protein 1 (*IGF2BP1*) expression [158].

let-7 (lethal-7) miRNA family is classified as tumor suppressive, since its members attenuate cancer aggressiveness, chemo- and radioresistance, but they result significantly reduced in GBM and MB CSCs [159,160,161,162]. Forced expression of let-7a can silence Kirsten rat sarcoma viral oncogene homolog (*KRAS*) and its downstream pathways PI3K/AKT and mitogen-activated protein kinase (MAPK)/ERK, inducing apoptosis in GSCs. Indeed, among the many oncogenes and miRNAs identified as regulators of cell growth/proliferation/apoptosis, *MYC* and let-7a have emerged as the respective key oncogene and miRNA deregulated in GSCs. let-7a has been shown to be part of the lin-28 homolog A (LIN28)/let-7/c-MYC triad, controlled by double-negative autoregulatory loops (LIN28/let-7 and Myc/let-7), which play a critical role in controlling apoptosis [248]. *LIN28* is a master regulator of pluripotency in embryonic stem cells (ESCs). In association with *NANOG*, *OCT4*, and *SOX2*, it is able to reprogram differentiated cells to pluripotent stem cells [249]. Moreover, let-7e suppresses the expression of nuclear paraspeckle assembly transcript 1 (NEAT1), a long non-coding RNA (lncRNA) that leads to enhanced apoptosis in U87 and U251 GSCs [160]. However, in uncommon situations, let-7 can act as an oncogene, reducing mitochondrial apoptosis and increasing cell proliferation, chemoresistance, tumor progression and metastasis [162]. Inhibition of let-7a-3p in U87 and U251 GSCs has been demonstrated to partly recover mitochondrial apoptosis induced by neurotensin receptor 1 (NTSR1) inhibition, along with downregulation of Bcl-2-like protein 2 (Bcl-w) and Bcl-2 [250]. The dual/two-faced behavior of let-7 indicates that its function may depend on specific target transcripts. Thus, further research studies are required to focus on the specific biological context of let-7 expression and its role in GSCs.

let-7a miRNA also acts as a tumor suppressor in NB by inhibiting oncogene transcription and stemness features of tumor cells [164]. It has been found to be downregulated in N- and I-type NB cell lines [243,244,245], likely due to its negative regulation by the neural crest-directed expression of LIN28 [251]. Therefore, difluoromethylornithine (DFMO) treatment on SMS-KCNR and BE(2)-C stem-like cells, characterized by V-Myc avian myelocytomatosis viral oncogene neuroblastoma (*MYCN*) amplification, reduces LIN28 and MYCN protein levels; thus, restoring let-7 miRNA expression and decreasing neurosphere formation [163,164].

### 3.2. Anti-Apoptotic miRNAs

Several miRNAs with anti-apoptotic function are upregulated in neural/neural crest CSCs. By controlling multiple target mRNAs, they suppress apoptotic pathways and ultimately preserve stem cell self-renewal and pluripotency.

In CD133+ GSCs, miR-9 is upregulated, negatively controlling the tumor suppressor gene Patched 1 (*PTCH1*) and resulting in diminished cell death. Moreover, miR-9 has been found to induce upregulation of the ATP binding cassette subfamily B member 1 (*ABCB1*) gene, leading to chemoresistance to TMZ. Knockdown of miR-9, instead, restores TMZ-induced cell death. In contrast, miR-9* (miR-9-3p) is downregulated in GSCs, although, when re-expressed, it can inhibit *SOX2*, a factor that confers self-renewal ability and drug resistance to GSCs [70,165,166,167,168].

miR-10b is upregulated in GSC lines, acting as a tumor promoter. Specifically, it has been reported to sustain cell stemness and affect the cell cycle, by targeting muscleblind-like splicing regulator 1 (*MBNL1-3*), spliceosome associated factor 3, U4/U6 recycling protein (*SART3*), arginine and serine rich coiled-coil 1 (*RSRC1*), and genes associated with RNA processing and splicing, according to Gene Set Enrichment Analysis (GSEA) [169,170]. Treatment with miR-10b inhibitors (anti-miR-10b) strongly impairs survival and proliferation and attenuates expression of stem cell markers nestin and OCT4. Moreover, miR-10b inhibition leads to the cleavage of CASP3 and CASP7, indicative of the induction of apoptotic cell death in GSCs [3,108,170].

Several studies have described miR-21 as one of the most consistently highly expressed oncomiRs in GBM, where it regulates multiple stemness parameters. Suppression of apoptosis is one of the key roles of miR-21. In GSCs, miR-21 confers resistance to apoptosis by targeting the *FASL* as well as by inhibiting an entire network of onco-suppressor genes, including *TP53*, *TGF-β*, *PTEN*, and programmed cell death 4 (*PDCD4*). Moreover, the high levels of miR-21 in U87 GSCs are able to prevent glioma cell apoptosis by inhibiting the tissue inhibitor of metalloproteinases 3 (*TIMP3*) gene, a suppressor of malignancy and inhibitor of matrix metalloproteinases (MMPs) [171]. Thus, inhibition of miR-21, by locked nucleic acid (LNA) or by anti-miR oligonucleotides, can not only restore apoptosis by activation of the caspase cascade via a FAS-dependent or PDCD4-mediated mechanism, but also stops cell cycle progression by decreasing EGFR/STAT3 signaling, and finally sensitizes GSCs to TMZ chemotherapy or radiation treatment [3,87,134,172,173,174].

Similarly, miR-21 has been found overexpressed in A375 melanoma stem-like cells, affecting cell apoptosis by targeting *TIMP3* [140,175,176,177].

miR-24, a member of the miR-23b cluster, is highly expressed in U87 GSCs, directly targeting suppression of tumorigenicity 7-like (*ST7L*). miR-24 abrogation allows *ST7L* restoration, which suppresses β-catenin/transcription factor 4 (TCF-4) activity, leading to cell cycle arrest at the G0/G1 phase and enhanced apoptosis. The same effect on the β-catenin/TCF-4 pathway, as well as reduced expression of the downstream *CCND1*, *STAT3* and *c-MYC*, is obtained by inhibition of miR-27b, another member of the miR-23b cluster, aberrantly upregulated in U87 GSCs [178,179].

miR-30 upregulation has been described as a key factor in the tumorigenicity of GSCs, by silencing suppressor of cytokine signaling 3 (*SOCS3*) and thereby enhancing the activation of the Janus kinase (JAK)/STAT3 signaling cascade, which sustains GSCs’ survival, proliferation, and antagonizes their differentiation. When a miR-30 inhibitor is transfected into U87 GSCs, the JAK/STAT3 signaling is significantly suppressed, as well as STAT3 phosphorylation [150,180].

The major tumor suppressor *PTEN* has been found to be targeted by several miRNAs, including miR-17-5p, -19a-3p, -19b-3p, belonging to the miR-17-92 cluster, and miR-21-5p, -26, -130b-3p, -221-3p, -222-3p, -494-3p, which are overexpressed in GSCs. They function as anti-apoptotic miRNAs by inhibiting *PTEN* and activating the PI3K/AKT signaling pathway as the main involved pathway [3,132,134,181,182].

miR-92b, upregulated in U87 GSCs, directly targets the SMAD family member 3 (*SMAD3*), a central activator of the TGF-β/cyclin-dependent kinase inhibitor 1A (CDKN1A/P21^CIP1^) apoptotic signaling pathway. On the contrary, the silencing of endogenous miR-92b has been reported to increase the expression of *SMAD3* and induce overexpression of P21, which halts cell cycle progression and warrants apoptosis [183].

miR-138 overexpression is considered a molecular signature of GSCs, by acting as a pro-survival oncomiR, which regulates multiple genes and pathways simultaneously, including bladder cancer associated protein (*BLCAP*), CASP3, nerve growth factor-inducible anti-proliferative (*BTG2*), and tumor suppressor candidate 2 (*TUSC2*). However, specific depletion of miR-138 in U87 and U251 cell lines can prevent cell survival and the formation of tumor spheres [126,184].

miR-149 overexpression in U87 GSCs has been related to a significant downregulation of caspase-2 (*CASP2*) expression and a downstream effect on p53 and p21 expression, leading to cell survival and proliferation [185,186].

It has been reported that the level of miR-154 in GBM is elevated and inversely correlated to the expression of its target phosphoribosyl pyrophosphate synthetase 1 (*PRPS1*), acting as a positive regulator of proliferation in CD133+ GSCs. Conversely, knockdown of miR-154 remarkably suppresses GSCs’ proliferation [252].

miR-155 is an oncomiR aberrantly expressed in GSCs that impairs cell differentiation by suppressing caudal-type homeobox 1 protein (CDX1). miR-155 upregulation has been shown to significantly inhibit early and late apoptosis in U87 GSCs, by affecting its downstream target *PTEN*, the expression of CASP3 and CASP9, and the PI3K/AKT signaling pathway [187]. Similarly, miR-155-3p is overexpressed in U87 GSCs, acting as an oncomiR by targeting the transcription factor sine oculis homeobox 1 (*SIX1*), promoting cell proliferation and preventing apoptosis. However, introducing a miR-155-3p inhibitor in chemoresistant cells rescues SIX1 expression, and thus induces cell cycle arrest at the G1/S phase and enhances TMZ-induced apoptosis [3,188].

miR-182, a member of a miRNA cluster in chromosomal locus 7q31–34, is frequently described as amplified in A375 melanoma cell lines, acting as an oncomiR through the suppression of transcription factors forkhead box O3 (*FOXO3*) and microphthalmia-associated transcription factor (*MITF*). On the contrary, its downregulation triggers apoptosis and blocks invasion [140,189,190,191].

miR-196a is overexpressed in GSCs, exerting its oncogenic effect by suppression of NFKB inhibitor alpha (*IκBα*) and consequent upregulation of NF-κB along with related anti-apoptotic proteins. miR-196a-5p acts as anti-apoptotic miRNA by inhibiting the transcription factor forkhead box O1 (*FOXO1*) as its main target [3,192,193].

miR-204-5p and miR-211-5p have high expression levels in A375 melanoma cells resistant to the B-Raf Proto-Oncogene, Serine/Threonine Kinase (BRAF) inhibitor vemurafenib, compared to parental cells. This overexpression leads to the inhibition of the proapoptotic transcription factor C/EBP homologous protein 10 (*CHOP*), also known as DNA damage-inducible transcript 3 (*DDIT3*) and as growth arrest and DNA damage-inducible protein 153 (*GADD153*), and insulin-like growth factor 2 receptor (*IGF-2R*), both recognized as miR-211-5p targets, and determines pro-survival cell responses in A375 cells [194,195].

miR-210 expression is elevated in GSCs, particularly in response to hypoxia conditions, in which it targets several genes such as MAX network transcriptional repressor (*MNT*). Knockdown of miR-210, by a specific antisense sequence, rescues the expression of *MNT*; thus, strongly prompting apoptosis, differentiation, and G0/G1 cell cycle arrest in hypoxic GSCs [3,186,196]. Moreover, miR-210 depletion causes a reduction in HIF1α protein expression, most likely due to the disruption of a hypoxia-induced positive feedback loop between HIF1α and miR-210, which represses glycerol-3-phosphate dehydrogenase 1-like (*GPD1L*), responsible for the HIF1α protein stability [196,197]. Regulator of differentiation 1 (*ROD1*) has been described as a further target for miR-210. Indeed, restoring ROD1 expression, by downregulation of miR-210 in U87 and U251 GSCs, inhibits cell proliferation and induces apoptosis [198].

miR-221 and -222 are upregulated in GSCs, both repressing apoptosis and prompting cell cycle progression by directly targeting the cell growth-suppressive *P27^KIP1^* and cyclin-dependent kinase inhibitor 1C (*CDKN1C/P57^KIP2^*) genes [132,199,253]. Furthermore, miR-221/222 cluster overexpression has been reported to unset apoptosis in GSCs by targeting p53-upregulated modulator of apoptosis (*PUMA*), which is not able to bind Bcl-2 and Bcl-2-like protein 1 (Bcl-x) anymore [200,201]. In this context, treatment of U87 and U251 GSCs with antisense oligonucleotides for miR-221/222 leads to cell cycle arrest in the G0/G1 phase as well as to enhanced sensitivity to radiotherapy and to TMZ, by regulating apoptosis independently of p53 status [199,201,202].

miR-221 and -222 have also been reported to be upregulated in A375 melanoma stem-like cells, resulting in reduced levels of their target V-Kit Hardy-Zuckerman 4 feline sarcoma viral oncogene (*C-KIT/CD117*), a melanocytic functional player with tumor-suppressive activity via apoptosis induction, occurring when its kinase activation is silenced [204]. Another proto-oncogene, targeted by miR-222, is V-Ets avian erythroblastosis virus E26 oncogene homolog 1 (*ETS-1*), involved in the induction of apoptosis [203,205]. Moreover, some studies have shown that miR-221/222 directly target *P27*, frequently downregulated in melanoma, which serves as a crucial cell cycle regulator through modulation of cyclin D1 [140,206,207].

miR-330, -363, and -582-5p, overexpressed in CD133+ GSCs, have been shown to function as pro-survival oncomiRs by inhibiting cell apoptosis through the silencing of the major performer caspases CASP3, CASP9, and Bcl-2-interacting mediator of cell death (BIM) signaling [87,208].

miR-335 is upregulated in U87 and U251 GSCs, due to amplification of its locus on chromosome 7q32, which confirms that chromosomal abnormalities and/or epigenetic events contribute to miRNA dysregulation. miR-335 function, by targeting the tumor suppressor disheveled-associated activator of morphogenesis 1 (*DAAM1*) and the transcription factor paired box 6 (*PAX6*), results in a pro-survival effect. Conversely, inhibition of miR-335 can rescue apoptosis and suppress cell growth [209,210,211].

In the NBL-WS human NB cell line, high expression levels of miR-380-5p have been related to inhibited *TP53* and activated Harvey rat sarcoma viral oncogene homolog (*HRAS*) genes. Conversely, as reported by Li et al. [212], the inhibition of miR-380-5p recovers p53 expression and apoptotic cell death, which implies the enhancing effect of miR-380-5p on neural CSCs [213].

miR-381 is a highly expressed oncomiR, which confers stemness, prompts tumor progression, and induces drug resistance in GBM cells. However, its activity can be suppressed by administration of antisense oligonucleotides, in combination with TMZ treatment [254].

A cluster including 14 miRNAs on the X chromosome, namely miR-506-514 cluster, is consistently overexpressed in A375 melanoma stem-like cells. Conversely, the inhibition of this cluster has been demonstrated to strongly enhance the percentage of apoptotic cells [255].

miR-638 has been shown to be upregulated during melanoma initiation and progression. By the marked reduction in the TP53-inducible nuclear protein 2 (*TP53INP2*) gene, it can suppress p53-mediated apoptosis pathways in A375 melanoma stem-like cells [214].

There is evidence that miRNAs play a key role in regulating the activation status of the apoptotic Hippo pathway via the post-transcriptional suppression of multiple target mRNAs of Hippo signaling components. Just as an example, the upregulation of miR-130b has been found to inactivate the Hippo pathway and promote CSC characteristics in GBM cells by directly suppressing macrophage stimulating 1/2 (*MST1/2*) and protein salvador homolog 1 (*SAV1*). Conversely, inhibition of miR-130b attenuates these effects, enabling the MST1/2-SAV1 kinase complex to activate the Hippo kinase apoptotic cascade [215].

Recent evidence supports the importance of hypoxic conditions in GSCs’ maintenance. Restricted oxygen levels have been shown to expand and preserve the fraction of such CSCs, thanks to the expression of both HIF1α/HIF2α subunits, which regulate the transcription of hundreds of genes in response to low levels of oxygen. HIF1α is a key regulator of CSC proliferation and fate in MB and GBM by activating the NF-κB pathway to sustain CSC survival and tumorigenesis [150,256,257]. In U251 GSCs, hypoxia requires Notch pathway activation to drive stemness maintenance [258,259]. The hypoxic niche also protects GSCs by limiting drug penetration and thus contributes to chemotherapy resistance [260]. Hypoxia and the stem-like state have been reported to influence the biogenesis and expression of a set of miRNAs, including miR-7-5p, -21-5p, -23b-3p, -205-5p, -210, and -373. At the same time, the dysregulation of these miRNAs can play a pro-survival role in this microenvironment, sustaining GSC stemness [3,108,196].

### 3.3. Pro- and Anti-Apoptotic miRNAs and Their Therapeutic Implication in Tumors

Current chemo- and radiotherapy treatments may be able to kill the bulk of tumor cells, but spare the relevant fraction of CSCs, which are protected by peculiar niches in the tumor mass and specific resistance mechanisms [261]. Since CSCs are the “seeds” for tumor initiation and development, metastasis, drug resistance, and recurrence, it is more than feasible that the development of therapeutic strategies directed to the CSCs- or CSC-like-subpopulations can be promising, especially if targeting those miRNAs involved in the regulation of CSC biology [150].

Many efforts are being made to knock out CSCs in tumors of neural/neural crest origin. Among the biological features of CSCs useful in the development of anti-CSC therapies, several chemotherapeutic drugs target the components of apoptotic pathways [75]. The balance between cell survival and apoptosis is crucial in CSCs, where the axis survival/cell cycle/apoptosis is dysregulated [114]. Deregulation of miRNAs in CSCs is heavily connected to apoptosis evasion, which leads to enhanced tumorigenesis and drug resistance [76]. Targeting the specific pattern of miRNAs that prevent neural CSCs from proceeding to apoptosis is, thus, emerging as an effective antitumor treatment and an adjuvant to standard therapies. Moreover, eliminating the CSC subpopulation, which survives over the conventional therapies, may improve the tumor management in clinical settings (Table 2).

Recently, it has been shown that HIFs are required for GSC maintenance and resistance to chemotherapy. Specifically, even under modest hypoxic conditions, *HIF2α* is elevated in GSCs, while it is not found in NSCs at mRNA or protein level. Hypoxia ensures resistance to TMZ as well, by inducing O6-methylguanine-DNA-methyltransferase (MGMT) expression in GSCs. TMZ treatment indeed has been shown incapable of halting self-renewal of CD133+ GSCs expressing MGMT [108,260]. For this reason, miRNAs modulating *HIF* expression in the hypoxic niche can offer hope as therapeutic targets in GBM to regulate the hypoxic HIF switch, the hypoxic microenvironment, and the consequent stem cell survival and resistance to therapy.

Therapeutic strategies based on targeting dysregulated miRNAs in CSCs include: the delivery of RNA oligonucleotides (miRNA mimics), designed to mimic endogenous CSC-suppressive miRNAs, downregulated in the CSC subpopulation; the introduction of antisense oligonucleotides (antagomiRs), with the aim to inhibit oncogenic miRNAs with CSC-enhancing function, overexpressed in CSCs; the regulation of CSC-related miRNAs by the administration of synthetic or natural agents. Both miRNA mimics and antagomiRs, thus, can control the expression of multiple genes and restore the signaling pathways leading to CSC death, resulting in a decrease in tumor progression and/or a sensitization to therapy.

Recently, nanotechnology has been applied to the design of lipid-based nanovectors, such as liposomes and lipoplexes, able to deliver chemically engineered sense or antisense oligonucleotides (Figure 1D). Moreover, intravenous delivery of tumor suppressing miRNAs or antagomiRs has been found to significantly induce apoptosis, as well as inhibit cell growth and proliferation in CSCs. Furthermore, a magnet-bead based miRNA delivery system has been developed. This system allows magnetic guidance to the site of interest and enables efficient miRNA delivery with very low cytotoxic effects. Further, exosomes can function as ideal drug carriers for antitumor therapies thanks to the many advantages, including low immunogenicity, biocompatibility, easy production, cytotoxicity, easy storage, high drug loading capacity, and long half-life [150].

Since the carrying capability is an index of the efficacy of nanomedicine, the design of efficient nanocarriers represents the current challenge necessary to improve the cellular intake and enhance the capability of entering within CSCs [71]. Meanwhile, the synthesis of specific and highly stable sense/antisense oligonucleotides is crucial to avoid off-target effects and to prevent their degradation during the delivery towards the target site, respectively.

At the same time, biotechnology has adopted the potential of RNA interference to knockdown the expression of specific RNAs and this option is being introduced in the clinic as a form of miRNA-based therapy. The advantage of miRNA administration as a cancer therapy is that a single miRNA can influence many pathways simultaneously to amplify its effect and finally achieve clinical benefit [83].

Distinct miRNAs are critical in the control of neural/neural crest-derived CSC survival. Therefore, in the context of a pro-apoptotic treatment, targeting their dysregulated expression to eradicate the tumor CSC population is emerging as a promising approach to cancer therapy. Dysregulation of miRNA expression is a mechanism that confers CSCs the ability to escape from apoptotic signals. Therefore, since apoptosis is the major type of cell death triggered by antitumor treatments [75], defective apoptosis may lead to drug resistance and then treatment failure and tumor relapse. Accordingly, due to their chemo- and radioresistant potential, GSCs, MB- and NB-CSCs, as well as melanoma stem cells, appear to be a powerful cellular target for such antitumor therapeutic regimens.

Specifically, GSC-targeted therapy is becoming a hot spot of recent studies for the treatment of glioma. The knowledge of miRNA expression levels and their correlation with GBM pathogenesis has led to the identification of specific miRNAs in GSCs with potential therapeutic applicability [277,278,279].

A novel multimodal therapy approach for GBM consists of oncomiR silencing, mediated by tumor-targeted nanoparticles, combined with standard chemo- and radiotherapeutic agents. This strategy has been applied in vitro combining miR-10b and miR-21 silencing with TMZ [262,263,264], or miR-21 inhibitors with the tyrosine kinase inhibitor sunitinib [265]. Similarly, treatment of glioma initiating cells (GICs) with NPV-LDE-225 (Erismodegib) has been shown to regulate proliferation and apoptosis, by means of the suppression of miR-21 [266,267]. Meanwhile, chemo- and radiotherapy have been reported to be involved in modulating the expression of miRNAs responsible for GBM development. It has been described that, after 48 h of treatment with TMZ and ionizing radiation, GSC neurospheres express higher levels of miR-21 in comparison to attached cells, confirming the crucial role of GSCs in the acquisition of chemo- and radioresistance [280].

Repression of the miR-381/neurofilament light polypeptide (NEFL) axis sensitizes GBM cells to TMZ-induced apoptosis by regulating multidrug resistance factors. Thus, this miRNA may potentially serve as a novel adjuvant therapeutic target for GBM [254].

Delivery of let-7a miRNA to GSCs has been carried out by magnetofection, using zinc-doped Fe_3_O_4_ nanoparticles, which show high cellular uptake (nearly 98%) and effective downregulation of the let-7a targets, *KRAS* and *PI3K* [281]. Moreover, a combined treatment of GSCs with phenformin and TMZ or dichloroacetate (DCA) succeeds in exerting an enhanced pro-apoptotic effect in vitro, by means of an increased expression and bioavailability of let-7, along with a marked inhibition of its target HMGA2 [269].

Treatment with miR-146b-5p, -181a, -211, and -224 mimics has been demonstrated to increase radiosensitivity of GSCs. Thus, these findings suggest that these miRNAs can provide new rationales for novel combinational therapies, in which they synergistically cooperate with standard treatments designed for GBM patients [128,134,139,270]. Moreover, the combination of radiotherapy and miR-153 ectopic expression in GSCs has been demonstrated to enhance the efficiency of glioma treatment [271].

miR-145 has been introduced as a novel therapeutic target for glioma in combination with the curcuminoid drug named demethoxycurcumin (DMC). Indeed, Lenti-GFP-miR-145 pretreatment increases GSC apoptosis induced by DMC, as demonstrated by both terminal deoxynucleotidyl transferase (TdT) dUTP nick- end labeling (TUNEL) cell apoptosis and histone–DNA enzyme-linked immunosorbent assay (ELISA) assays [126].

The exposure to curcumin (diferuloylmethane), a natural compound extracted from turmeric (*Curcuma longa* Linn.), can lead to upregulation of miR-146a, which significantly silences the transcriptional activity of NF-κB in U87 GSCs. Thus, TMZ-induced cytotoxicity can be potentiated by the concomitant treatment with curcumin [272].

Treatment with miR-326 mimics exerts a tumor inhibition effect by prompting curcumin-mediated apoptosis, by means of decreasing the activity of the SHH/GLI1 pathway. Considering the GLI1-p53 functional network, increased p53 expression occurs in U87 (p53 wild-type) but not in U251 GSCs (p53 mutant), suggesting that miR-326 and curcumin combinatorial therapy can decrease SHH/GLI1 activity also independently of the p53 status. In addition, the increased miR-326 expression, observed in response to curcumin treatment, underlines the feedback loop between miR-326 and the SHH/GLI1 signaling, providing a mechanism for the enriched anti-glioma effect due to the combined treatment [273]. Taken together, these findings support the suggestion by Klinger et al. about the need for clinical trials with curcumin for the treatment of GBM and other brain tumors [267,282].

The combined treatment with miR-125b inhibitor and PI3K/Akt pathway inhibitor LY294002 has been shown to reverse the chemoresistance of GSCs and then to sensitize these cells to TMZ-induced apoptosis by targeting the Wnt/β-catenin signaling pathway [150,268].

An increasing number of studies has shown that elevated levels of distinct oncomiRs, such as miR-10b [169,264,283], -30 [180], -221/222 [284], -335 [285], are associated with poor survival in GBM patients, according to Kaplan–Meier survival curves and referring to The Cancer Genome Atlas (TCGA) data. This is why the modulation of such miRNAs’ activity by antisense molecules appears to be a promising therapeutic option.

Within the framework of the development of effective therapies for the treatment of NB, the manipulation of miRNA expression and the induction of apoptosis and differentiation in the CSC subpopulation seem to be the most promising strategies investigated in the last few decades [80,263,286,287].

The resistance of melanoma to conventional therapies is, at least in part, attributed to its CSC population [274]. Thus, targeting specific miRNAs involved in the regulation of the CSC-associated chemo- and radiotherapy-resistant phenotype could be useful for the preferential killing of CSCs, recovering therapy sensitivity.

In the context of melanoma treatment, novel epigenetic strategies using miRNAs derived from natural food have been proposed. Indeed, the transfection of melanoma stem cells with cross-phylum-derived miRNAs has been demonstrated to be effective in the induction of apoptosis, as well as in the suppression of cell stemness, viability, and proliferation. The shrimp-derived miR-S8 has been described to suppress stemness and tumor progression by degrading the mRNA of the transcription factor human Y-box binding protein 1 (*YB-1*) in melanoma stem cells [275]. Likely, mja-miR-35-3p, which belongs to the shrimp *Marsupenaeus japonicus* and possesses antiviral activity in this species, has been demonstrated to induce cell cycle arrest in the G1 phase to boost apoptosis, and to impair the sphere-forming capacity of melanoma stem cells, by suppressing the protein interacting with never in mitosis A1 (*PIN1*) gene [276]. In the same way, the shrimp miR-965 can suppress the stemness and proliferation of these cells by interacting with the human eukaryotic translation initiation factor 2C 2 (Ago2) protein and suppressing myeloid cell leukemia sequence 1 (Bcl2-related) (*MCL1*) expression [274]. Taken together, these studies suggest an alternative mechanism of CSC regulation by miRNAs in a cross-species manner, which can represent a promising natural drug in the treatment of CSC-driven tumors.

Overall, clinical trials exploiting miRNAs as therapeutic targets in the treatment of GBM and melanoma have been extensively described by Chakraborty et al. [288] and Rupaimoole and Slack [289], and the updates on following clinical trial phases have been reported by more recent studies [102,290,291,292].

Targeting of CSCs by all these forms of miRNA modulation has been claimed to be promising for cancer treatment since it could overcome the limitations of conventional non-specific antitumor therapies, such as chemo- and radiotherapy. In this way, in combination with standard therapies, addressing dysregulated miRNAs to eliminate the aggressive and drug-resistant CSC population could effectively inhibit tumor progression, metastasis, recurrence, and therapy resistance to achieve better treatment outcomes. In light of this, refining the knowledge regarding the miRNA expression pattern in CSCs harbored in tumors of neural/neural crest origin will identify novel specific therapeutic targets against these cancers, paving the way for antitumor personalized medicine.

## Figures and Tables

**Table 1:** Pro- and anti-apoptotic miRNAs associated with apoptosis regulation in CSCs of neural/neural crest origin.

miRNA	Target mRNA/Pathway	miRNA Effect on Apoptosis	miRNA Expression in CSCs	Cancer Type	Ref.
miR-7	*EGFR, AKT, DR5*	↑	down	glioma	[83]
miR-23b	*HMGA2*	↑	down	glioma	[84]
miR-26a	*SODDs*	↑	down	melanoma	[85,86]
miR-29a	*QKI-6*	↑	down	glioma	[87,88]
miR-34a	*BCL2, TP53, NOTCH1/NOTCH2, Dll1, RICTOR, AKT,* Wnt/β-catenin	↑	down	glioma, MB	[89,90,91,92]
	*TP53, NOTCH*	↑	down	melanoma	[79,93,94,95]
miR-107	*NOTCH2, MMP-12*	↑	down	glioma	[96]
miR-124	*CDK6, RB*	↑	down	glioma	[4,97]
miR-137	*CDK6, RB, GLIPR*	↑	down	glioma	[4,97,98,99]
	*CTBP1, PIK3R3*	↑	down	melanoma	[100,101,102]
miR-125a-5p	*LIN28B*	↑	down	melanoma	[103,104]
miR-125b	*CDK6, CDC25A*	↑	down	glioma	[105,106,107,108]
	*TNFAIP3, NKIRAS2, TP53, p38MAPK, BAK1*	↓	up	glioma	[109,110,111]
	*TP53*	↓	up	NB	[112,113]
	*NEDD9*	↓	up	melanoma	[114]
miR-128	*EGFR/PDGFR/AKT, BMI-1*	↑	down	glioma	[105,115,116,117]
miR-218	*CDK6, MKI67, ECOP*	↑	down	glioma	[108,118,119,120]
miR-129-5p	*FNDC3B*	↑	down	glioma	[121]
miR-134	*NANOG*	↑	down	glioma	[122]
miR-138	*HIF1α*	↑	down	melanoma	[123]
miR-141	*JAG1*	↑	down	glioma	[124]
miR-145	*BNIP3, NOTCH, SOX9, ADD3*	↑	down	glioma	[125,126,127]
miR-146b-5p	*HuR*	↑	down	glioma	[128]
miR-149	*BCL2, CDC42*	↑	down	NB	[129]
miR-152/153	*KLF4, LGALS3*	↑	down	glioma	[105,130,131,132]
miR-181	*NOTCH2*	↑	down	glioma	[133,134]
miR-182	*BCL2L12, c-MET, HIF2α*	↑	down	glioma	[135]
miR-199b-5p	*HES1*	↑	down	MB	[136,117]
miR-200b	*CD133*	↑	down	glioma	[137]
miR-203	*PLD2*	↑	down	glioma	[138]
miR-211	*MMP-9, BCL2*	↑	down	glioma	[139]
	*KCNMA1*	↑	down	melanoma	[140,141,142,143]
miR-218	*CIP2A, BMI-1*	↑	down	melanoma	[102,144]
miR-219-5p	*BCL2*	↑	down	melanoma	[145]
miR 302-367	*CXCR4, CXCL12*	↑	down	glioma	[146]
miR-326	*NOTCH, SMO, GLI1*	↑	down	glioma, MB	[108,117,147,148,149,150]
miR-340	*ROCK1*	↑	down	glioma	[151]
miR-451	*AKT1, CCND1, MMP-2, MMP-9, BCL2, CAB39*	↑	down	glioma	[152,153,154]
miR-503	*IGF-1R/PI3K/AKT*	↑	down	glioma	[155]
miR-608	*MIF*	↑	down	glioma	[3,156]
miR-625	*SOX2*	↑	down	melanoma	[104,157]
miR-873	*IGF2BP1*	↑	down	glioma	[158]
let-7	*KRAS, PI3K/AKT, MAPK/ERK, LIN28/*let-7*/c-MYC, NEAT1*	↑	down	glioma, MB	[159,160,161,162]
	*MYCN*	↑	down	NB	[163,164]
miR-9	*PTCH1*	↓	up	glioma	[71,165,166,167]
miR-9*	*SOX2*	↑	down	glioma	[168,165]
miR-10b	*MBNL1-3, SART3, RSRC1*	↓	up	glioma	[3,108,169,170]
miR-21	*FASL, TP53, TGF-β, PTEN, PDCD4, TIMP3*	↓	up	glioma	[3,87,134,171,172,173,174]
	*TIMP3*	↓	up	melanoma	[140,175,176,177]
miR-24	*ST7L,* β-catenin*/TCF-4*	↓	up	glioma	[178]
miR-27b	β-catenin*/TCF-4*	↓	up	glioma	[179]
miR-30	*SOCS3, JAK/STAT3*	↓	up	glioma	[150,180]
miR-17-92	*PTEN, PI3K/AKT*	↓	up	glioma	[3,132,134,181,182]
miR-92b	*SMAD3, TGF-β/P21*	↓	up	glioma	[183]
miR-138	*BLCAP, CASP3, BTG2, TUSC2*	↓	up	glioma	[126,184]
miR-149	*CASP2*	↓	up	glioma	[185,186]
miR-155	*PTEN, CASP3, CASP9, PI3K/AKT, CDX1, SIX1*	↓	up	glioma	[3,187,188]
miR-182	*FOXO3, MITF*	↓	up	melanoma	[140,189,190,191]
miR-196a	*IκBα*	↓	up	glioma	[192]
miR-196a-5p	*FOXO1*	↓	up	glioma	[193]
miR-211–5p	*CHOP, IGF-2R*	↓	up	melanoma	[194,195]
miR-210	*MNT,HIF1α/*miR-210*/GPD1L, ROD1*	↓	up	glioma	[186,196,197,198]
miR-221/222	*P27, P57, PUMA*	↓	up	glioma	[199,200,201,202]
	*C-KIT, ETS-1, P27*	↓	up	melanoma	[140,203,204,205,206,207]
miR-330, -363, -582-5p	*CASP3, CASP9, BIM*	↓	up	glioma	[87,208]
miR-335	*DAAM1, PAX6*	↓	up	glioma	[209,210,211]
miR-380-5p	*TP53, HRAS*	↓	up	NB	[212,213]
miR-638	*TP53INP2*	↓	up	melanoma	[214]
miR-130b	*MST1/2, SAV1,* Hippo pathway	↓	up	glioma	[215]

↑ pro-apoptotic; ↓ anti-apoptotic; down: downregulated; up: upregulated; MB: medulloblastoma; NB: neuroblastoma.

**Table 2:** miRNA-based experimental antitumoral therapeutic approaches.

Therapeutic Agent	Combined Treatment	Cancer Type	Ref.
**antagomiR**			
anti-miR-381	TMZ	GBM	[254]
anti-miR-10b + anti-miR-21	TMZ	glioma, GBM	[262,263,264]
anti-miR-21	Sunitinib	GBM	[265]
anti-miR-21	Erismodegib	glioma	[266,267]
anti-miR-125b	LY294002 + TMZ	GBM	[268]
**miRNA mimics**			
miR-145	DMC	GBM	[126]
miR-146b-5p	Radiotherapy	glioma	[128]
miR-181a	Radiotherapy	GBM	[134]
miR-211	Radiotherapy + TMZ	GBM	[139]
let-7	Phenformin + TMZ/DCA	glioma	[269]
miR-224	Radiotherapy	GBM	[270]
miR-153	Radiotherapy	GBM	[271]
miR-146a	Curcumin + TMZ	GBM	[272]
miR-326	Curcumin	GBM	[273]
**Cross-phylum derived miRNAs**			
Shrimp-derived miR-S8, -35-3p, -965		melanoma	[274,275,276]

TMZ: temozolomide; DCA: dichloroacetate; DMC: demethoxycurcumin; GBM: glioblastoma multiforme.

## Abbreviations

Aβ	amyloid β-peptide
ABC	ATP binding cassette transporter
*ABCB1*	ATP binding cassette subfamily B member 1
AD	Alzheimer’s disease
*ADD3*	adducin 3
ADSC	adipose-derived stem cell
*Ago2*	eukaryotic translation initiation factor 2C 2
AIS	acute ischemic stroke
*AKT*	serine-threonine protein kinase
ALS	amyotrophic lateral sclerosis
ApoE	apolipoprotein E
ASD	autism spectrum disorder
*BAK1*	Bcl-2 antagonist/killer 1
*Bcl-2*	Bcl-2 apoptosis regulator
*BCL2L12*	Bcl-2-like protein 12
*Bcl-w*	Bcl-2-like protein 2
*Bcl-x*	Bcl-2-like protein 1
BH3	Bcl-2 homology 3 domain
*BIM*	Bcl-2-interacting mediator of cell death
*BLCAP*	bladder cancer associated protein
*BMI-1*	BMI1 proto-oncogene, polycomb ring finger
BMSC	bone marrow mesenchymal stem cell
*BNIP3*	Bcl-2 interacting protein 3
*BRAF*	B-Raf Proto-Oncogene, Serine/Threonine Kinase
*BTG2*	nerve growth factor-inducible anti-proliferative
BTSC	brain tumor stem cell
*CAB39*	calcium binding protein 39
*CASP2*	caspase-2
*CASP3*	caspase-3
*CASP7*	caspase-7
*CASP9*	caspase-9
*CCND1*	cyclin D1
*CD133*	prominin-1
*CDC25A*	cell division cycle 25A
*CDC42*	cell division cycle 42
*CDK6*	cyclin-dependent kinase 6
*CDKN1A/P21^CIP1^*	cyclin-dependent kinase inhibitor 1A
*CDKN1B/P27^KIP1^*	cyclin-dependent kinase inhibitor 1B
*CDKN1C/P57^KIP2^*	cyclin-dependent kinase inhibitor 1C
*CDX1*	caudal-type homeobox 1 protein
*c-FLIP*	cellular FLICE-like inhibitory protein
*CHOP*	C/EBP homologous protein 10
*CIP2A*	cellular inhibitor of PP2A
*C-KIT/CD117*	V-Kit Hardy-Zuckerman 4 feline sarcoma viral oncogene
*c-MET*	MET proto-oncogene, receptor tyrosine kinase
*c-MYC*	myc proto-oncogene
CNS	central nervous system
CpG	cytosine-phosphate-guanine
CREB	cAMP response element-binding protein
CSC	cancer stem cell
CSF	cerebral spinal fluid
*CTBP1*	C-terminal binding protein 1
*CXCL12*	C-X-C motif chemokine ligand 12
*CXCR4*	C-X-C motif chemokine receptor 4
*DAAM1*	disheveled-associated activator of morphogenesis 1
DCA	dichloroacetate
*DDIT3*	DNA damage-inducible transcript 3
DFMO	difluoromethylornithine
*Dll1*	Notch ligand Delta-like 1
DMC	demethoxycurcumin
DOX	doxorubicin
*DR5*	death receptor 5
*E2F*	E2 factor family of DNA-binding transcription factor
*E2F2*	E2F transcription factor 2
*ECOP*	epidermal growth factor receptor-coamplified and overexpressed protein
*EGFR*	epidermal growth factor receptor
ELISA	enzyme-linked immunosorbent assay
EMT	epithelial-to-mesenchymal transition
*ERK 1/2*	extracellular regulated kinase 1/2
ESC	embryonic stem cell
*ETS-1*	V-Ets avian erythroblastosis virus E26 oncogene homolog 1
*FASL*	Fas cell surface death receptor ligand
*FNDC3B*	fibronectin type III domain containing 3B
*FOXO1*	forkhead box O1
*FOXO3*	forkhead box O3
*GADD153*	growth arrest and DNA damage-inducible protein 153
*GAP43*	growth associated protein 43
GBM	glioblastoma multiforme
*GFAP*	glial fibrillary acidic protein
GIC	glioma initiating cell
*GLI1*	glioma-associated oncogene homolog 1 (zinc finger protein)
*GLIPR*	glioma pathogenesis-related protein
*GPD1L*	glycerol-3-phosphate dehydrogenase 1-like
GSC	glioma stem cell
*GSEA*	Gene Set Enrichment Analysis
*HCELL/CD44*	hematopoietic cell E/L-selectin ligand
*HES1*	class B basic helix-loop-helix protein 39
*HIF1α*	hypoxia-inducible factor-1 alpha
*HIF2α*	hypoxia-inducible factor-2 alpha
hiPSC	human induced pluripotent stem cell
*HMGA2*	high mobility group AT-hook 2
*HRAS*	Harvey rat sarcoma viral oncogene homolog
*HuR*	Hu antigen R
IAP	inhibitor of apoptosis protein
*IGF-1R*	insulin-like growth factor-1 receptor
*IGF2BP1*	insulin-like growth factor 2 MRNA binding protein 1
*IGF-2R*	insulin-like growth factor 2 receptor
*IκBα*	NFκB inhibitor alpha
*JAG1*	jagged canonical Notch ligand 1
*JAK*	Janus kinase
*JNK*	c-Jun N-terminal kinase
*KCNMA1*	potassium calcium-activated channel subfamily M alpha 1
*KLF4*	Krüppel-like factor 4
*KRAS*	Kirsten rat sarcoma viral oncogene homolog
*LGALS3*	galectin 3
*LIN28*	lin-28 homolog A
*LIN28B*	lin-28 homolog B
LNA	locked nucleic acid
lncRNA	long non-coding RNA
*MAPK*	mitogen-activated protein kinase
MB	medulloblastoma
*MBNL1-3*	muscleblind-like splicing regulator 1
*MBP*	myelin basic protein
MCAO	middle cerebral artery occlusion
*MCL1*	myeloid cell leukemia sequence 1 (Bcl2-related)
MCSC	melanocyte stem cell
*MEK1/2*	mitogen-activated protein kinase kinase 1/2
mRNA	messenger RNA
*MGMT*	O6-methylguanine-DNA-methyltransferase
miRNA	microRNA
*MIF*	macrophage migration inhibitory factor
*MITF*	microphthalmia-associated transcription factor
*MKI67*	marker of proliferation Ki-67
MMP	matrix metalloproteinase
*MMP-2*	matrix metalloproteinase-2
*MMP-9*	matrix metalloproteinase-9
*MMP-12*	matrix metalloproteinase-12
*MNT*	MAX network transcriptional repressor
MS	multiple sclerosis
MSC	mesenchymal stem cell
*MST1/2*	macrophage stimulating 1/2
*mTOR*	mammalian target of rapamycin
mTORC2	mammalian target of rapamycin complex 2
*MYCN*	V-Myc avian myelocytomatosis viral oncogene neuroblastoma
*NANOG*	nanog homeobox
NB	neuroblastoma
*NEAT1*	nuclear paraspeckle assembly transcript 1
*NEDD9*	neural precursor cell expressed, developmentally down-regulated 9
*NEFL*	neurofilament light polypeptide
*NF*	neurofilament
*NF-κB*	nuclear factor kappa-light-chain-enhancer of activated B cells
*NKIRAS2*	NF-κB inhibitor interacting RAS-like 2
*NOTCH1/2*	Notch receptor 1/2
NPC	neural progenitor cell
NSC	neural stem cell
*NTSR1*	neurotensin receptor 1
*OCT4*	octamer-binding transcription factor 4
OPC	oligodendrocyte precursor cell
*p38MAPK*	p38 mitogen-activated protein kinase
*PAX6*	paired box 6
PD	Parkinson’s disease
*PDCD4*	programmed cell death 4
*PDGFR*	platelet-derived growth factor receptor
*PI3K*	phosphoinositide-3-kinase C
*PIK3R3*	phosphatidylinositol-3-kinase regulatory subunit 3
*PIN1*	protein interacting with never in mitosis A1
*PLD2*	phospholipase D2
*PRPS1*	phosphoribosyl pyrophosphate synthetase 1
*PTCH1*	Patched 1
*PTEN*	phosphatase and tensin homolog
*PUMA*	p53-upregulated modulator of apoptosis
*QKI-6*	Quaking gene isoform 6
*RELA*	RELA proto-oncogene, NF-KB p65 subunit
*RB*	retinoblastoma protein
*RICTOR*	rapamycin-insensitive companion of mTOR
rmTBI	repetitive mild traumatic brain injury
*ROCK1*	rho associated coiled-coil containing protein kinase 1
*ROD1*	regulator of differentiation 1
*RSRC1*	arginine and serine rich coiled-coil 1
*RTVP-1*	related to testis-specific, vespid, and pathogenesis proteins 1
*SART3*	spliceosome associated factor 3, U4/U6 recycling protein
*SAV1*	protein salvador homolog 1
SCI	spinal cord injury
Shh	sonic hedgehog
shRNA	short hairpin RNA
*SIX1*	sine oculis homeobox 1
*SMAD*	mothers against decapentaplegic homolog
*SMAD3*	SMAD family member 3
*SMO*	smoothened, frizzled class receptor
*SOCS3*	suppressor of cytokine signaling 3
*SODDs*	silencer of death domains
*SOX2*	sex determining region Y-box 2
*SOX9*	SRY-box transcription factor 9
*SSEA-1/CD15*	stage specific embryonic antigen 1
*ST7L*	suppression of tumorigenicity 7-like
*STAT3*	signal transducer and activator of transcription 3
TBI	traumatic brain injury
*TCF-4*	transcription factor 4
TCGA	The Cancer Genome Atlas
*TGF-β*	transforming growth factor-β
*TIMP3*	tissue inhibitor of metalloproteinases 3
TLR4	Toll-like receptor 4
TMZ	temozolomide
*TNFAIP3*	tumor necrosis factor alpha-induced protein 3
*TNFα*	tumor necrosis factor alpha
*TP53*	tumor protein p53
*TP53INP2*	TP53-inducible nuclear protein 2
*TRAIL*	tumor necrosis factor apoptosis inducing ligand
TUNEL	terminal deoxynucleotidyl transferase (TdT) dUTP nick- end labeling
*TUSC2*	tumor suppressor candidate 2
*Wnt*	wingless-related integration site
*WT1*	Wilms tumor protein 1
*YB-1*	human Y-box binding protein 1
